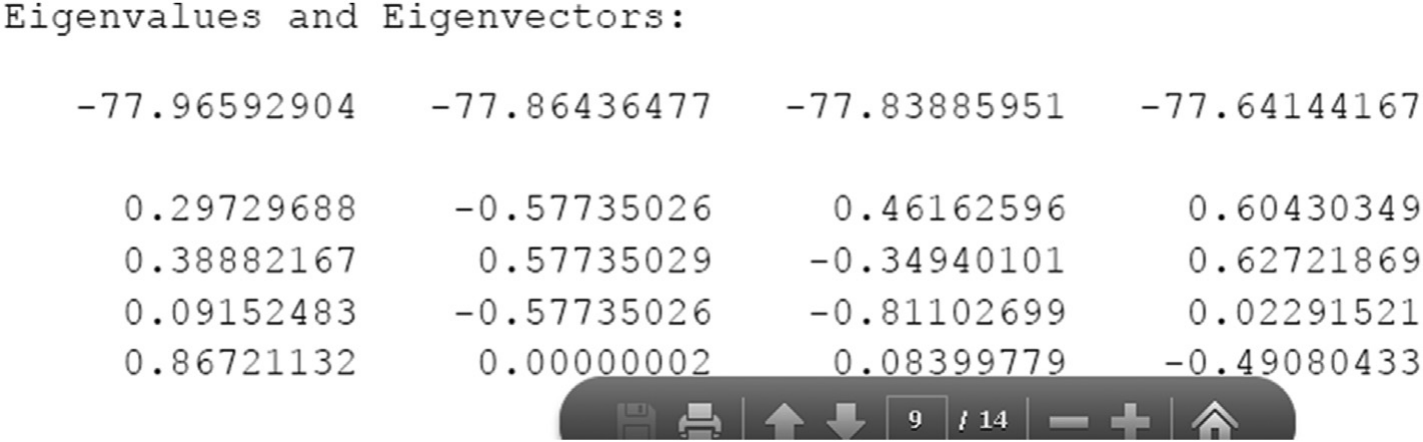# Source code, input data, and sample output concerning the application of multistate density functional theory to the singdoublet and tripdoublet states of the ethylene cation

**DOI:** 10.1016/j.dib.2019.104984

**Published:** 2019-12-13

**Authors:** Likun Yang, Adam Grofe, Jeffrey R. Reimers, Jiali Gao

**Affiliations:** a International Centre for Quantum and Molecular Structures and the Department of Physics, Shanghai University, Shanghai, 200444, China; b Laboratory of Theoretical and Computational Chemistry, Institute of Theoretical Chemistry, Jilin University, Changchun, Jilin Province, 130023, China; c School of Mathematical and Physical Sciences, University of Technology Sydney, NSW, 2007, Australia; d Shenzhen Bay Laboratory, Shenzhen, 518055, China; e Peking University Shenzhen Graduate School, Shenzhen, 518055, China; f Department of Chemistry and Supercomputing Institute, University of Minnesota, Minneapolis, MN, 55455, United States

**Keywords:** Multi-state density functional theory, Open shell, Spin contamination

## Abstract

This is the data and associated new software required to run multi-state density-functional theory (MSDDFT) calculations by the GAMESS programme. Also, data and software needed to drive GAMESS based on output from the Gaussian-16 package is included. Sample input and output files are included, as well as Perl scripts and Fortran source code. A separate execution of the scripts is required to create the input specifications for each state to be included in the MSDFT, then after GAMESS is run more software is included to calculate the final state energies. The associated basic theory and results are described in “Multistate density functional theory applied with 3 unpaired electrons in 3 orbitals: the singdoublet and tripdoublet states of the ethylene cation” [1].

**Table:** Specifications table

Subject	Physical and Theoretical Chemistry
Specific subject area	How to run multi-state DFT calculations, in particular on open-shell systems, with an example for ethylene cation excited states with 3 unpaired electrons in 3 orbitals
Type of data	Input and output files for software plus source code
How data were acquired	The source code and input control files were prepared by human construction. Some of the provided output files were prepared by running this source code using the provided data. These were then used as input for a (slightly modified version) of the GAMESS software package, the output of which is also included in the provided data.
Data format	all are Ascii text files, source code is Perl or Fortran, also one Excel spreadsheet.
Parameters for data collection	All data presented pertains to the situation in which 3 electrons are distributed in 3 orbitals. The provided software is expected to be of general use when applied to other situations, and indeed inclusion of an optional fourth state is also described. The presented sample data and results pertain to the ethylene cation as a model system.
Description of data collection	operation on Linux and Windows10 environments
Data source location	China and Australia
Data accessibility	With the article
Related research article	Likun Yang, Adam Grofe, Jeffrey R Reimers, Jiali Gao Multistate density functional theory applied with 3 unpaired electrons in 3 orbitals: the singdoublet and tripdoublet states of the ethylene cation Chemical Physics Letters https://doi.org/10.1016/j.cplett.2019.136803

**Table:** 

**Value of the Data** • This data allows MSDFT calculations to be run in GAMESS, with added interface to Gaussian16 • It will benefit anyone wishing to perform MSDFT calculations. • The data can be modified to accommodate any other molecular system.

## 1 Data

The data provided shows an implenetation of multi-state density-functional theory (MSDFT) [1] that interfaces the Gaussian16 [2] and GAMESS [3] software packages. It comes in four categories (1): files needed to run Gaussian16 (ethene-quartet-aug-cc-pvdz.com) and the output it produces (ethene-quartet-aug-cc-pvdz.log, ethylene-quartet.fchk and related file ethylene-ground-state.fchk); (2) source code to convert Gaussian16 output wavefunction files into GAMESS input wavefunction files (fchk-to-bmo-2a.pl, fchk-bmo-3a.pl, fchk-to-bmo-4a.pl, fchk-to-bmo-b2i-to-b3y.pl and fchk-to-bmo.f90); (3) files needed to run GAMESS (msdft-gamess-ci.inp) and its output (msdft-gamess-ci.out); and (4) source code to process the GAMESS output to produce the final energies (coupling-spreadsheet.xlsx and mod-ham.f90) and its input file mod-ham-aug-cc-pvdz.dat and produced output mod-ham-aug-cc-pvdz.out.

## 2 Experimental design, materials, and methods

A novel aspects of the methods allow for the porting of wavefunctions generated by common electronic-structure programmes implementing Kohn-Sham density-functional theory into the GAMESS program for use in MSDFT calculations. Alternatively, any software could be used that produced the desired output for the states of interest, including GAMESS itself. Provided here is software for the conversion of the Gaussian16 binary checkpoint file produced by Gaussian-16:

**Table:** 

*.pl	Perl scripts, converts the supplied Gaussian16 fchk files to the four required GAMESS bmo files
fchk-to-bmo.f90	Fortran module called by the Perl script, calls the BLAS library

and software needed to solve the MSDFT Hamiltonian to get the final energy levels:

**Table:** 

Coupling-spreadsheet.xlsx	Determines off-diagonal matrix elements from GAMESS energies
Mod-ham.f90	Determines final energies from the multi-state Hamiltonian

A slightly modified version of GAMESS is used, obtainable from jiali@jialigao.org. A full sequence of events pertinent to using Gaussan-16 orbitals to drive a GAMESS MSDFT calculation for the quartet and doublet states, plus one additional state, pertaining to 3 electrons in 3 orbitals is:

1. Use Gaussian16 to perform restricted open-shell (RO) DFT optimization of the quartet state, in the example this is (1)^4^B_2u_, with input file ethene-quartet-aug-cc-pvdz.com
Using the command “g16 ethene-quartet-aug-cc-pvdz.com”.
This produces the supplied output file: ethene-quartet-aug-cc-pvdz.log.
2. Format the Gaussian16 checkpoint file to make a.fchk file using command “formchk ethene-quartet-aug-cc-pvdz”. Then use the embedded orbitals to create GAMESS input.bmo files for the three-possible determinants containing two unpaired alpha electrons and one unpaired beta electron, named 2a, 3a and 4a. In addition, we include an optional 4th state which in this case arises from a B_2u_ to B_3u_ excitation
fchk-to-bmo-2a.pl ethene-quartet.fchk
fchk-to-bmo-3a.pl ethene-quartet.fchk
fchk-to-bmo-4a.pl ethene-quartet.fchk
fchk-to-bmo-b2u-to-b3u.pl ethene-ground-state.fchk
3. Use GAMESS to compute the MSDFT configuration interaction including all 4 states using command
“rungms msdft-gamess-ci.inp AG 1 > msdft-gamess-ci.out”. (Here ‘AG’ (Adam Grofe) means call for a special version of GAMESS that do MSDFT; ‘1’ means use one process).
4. Use the xlsx spreadsheet to compute the spin coupling using the energy differences between the 2a, 3a and 4a with the 3/2 state, entering the appropriate energies extracted from the GAMESS output:

**Figure:**
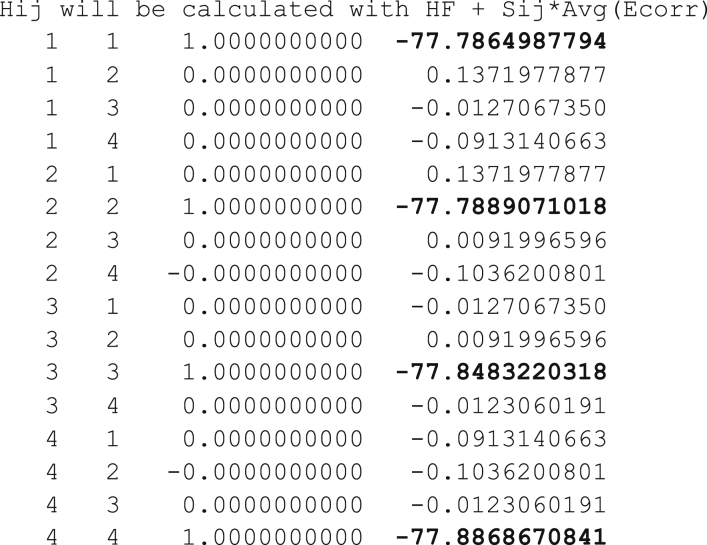

This step modifies the off-diagonal elements of the Hamiltonian to eliminate spin contamination, appropriate for 3 electrons in 3 orbitals. The data to be copied into the spreadsheet is the diagonal elements of the MSDFT Hamiltonian in the output of the last step. The xlsx file with its input and output then looks like:

**Figure:**
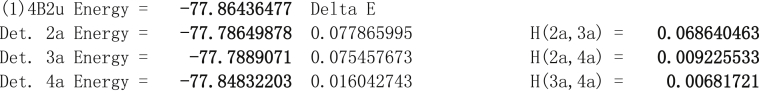

5. Modify the Hamiltonian to use the new couplings and compute the energies by using mod-ham.f90.
The command is “mod-ham.exe mod-ham-aug-cc-pvdz.dat”
The input data file is constructed by copying the full Hamiltonian matrix (reproduced above) from the GAMESS output into mod-ham-aug-cc-pvdz.dat, then adding the output energies from the as well as modified couplings obtained from the spreadsheet, changing the sign to be that from the original matrix:

**Figure:**
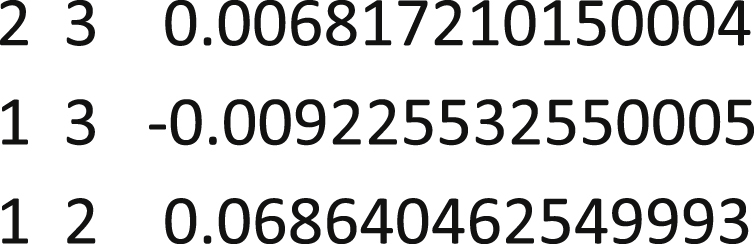

mod-ham.exe makes mod-ham-aug-cc-pvdz.out containing the final energies and eigenvectors

**Figure:**